# Development of the excretory system in a polyplacophoran mollusc: stages in metanephridial system development

**DOI:** 10.1186/1742-9994-9-23

**Published:** 2012-09-14

**Authors:** Natalie Baeumler, Gerhard Haszprunar, Bernhard Ruthensteiner

**Affiliations:** 1 Zoologische Staatssammlung München, Münchhausenstr. 21, 81247 München, Germany; 2 Department Biology I, Ludwig-Maximilians-Universität-München, Großhadernerstrasse 2, D-82152 Planegg-Martinsried, Germany

**Keywords:** Metanephridial system, Nephridia, Protonephridia, Coelomic cavities, Ontogeny, Homology, Mollusca, Polyplacophora

## Abstract

**Background:**

Two types of excretory systems, protonephridia and metanephridial systems are common among bilaterians. The homology of protonephridia of lophotrochozoan taxa has been widely accepted. In contrast, the homology of metanephridial systems – including coelomic cavities as functional units – among taxa as well as the homology between the two excretory systems is a matter of ongoing discussion. This particularly concerns the molluscan kidneys, which are mostly regarded as being derived convergently to the metanephridia of e.g. annelids because of different ontogenetic origin. A reinvestigation of nephrogenesis in polyplacophorans, which carry many primitive traits within molluscs, could shed light on these questions.

**Results:**

The metanephridial system of *Lepidochitona corrugata* develops rapidly in the early juvenile phase. It is formed from a coelomic anlage that soon achieves endothelial organization. The pericardium and heart are formed from the central portion of the anlage. The nephridial components are formed by outgrowth from lateral differentiations of the anlage. Simultaneously with formation of the heart, podocytes appear in the atrial wall of the pericardium. In addition, renopericardial ducts, kidneys and efferent nephroducts, all showing downstream ciliation towards the internal lumen, become differentiated (specimen length: 0.62 mm). Further development consists of elongation of the kidney and reinforcement of filtration and reabsorptive structures.

**Conclusions:**

During development and in fully formed condition the metanephridial system of *Lepidochitona corrugata* shares many detailed traits (cellular and overall organization) with the protonephridia of the same species. Accordingly, we suggest a serial homology of various cell types and between the two excretory systems and the organs as a whole. The formation of the metanephridial system varies significantly within Mollusca, thus the mode of formation cannot be used as a homology criterion. Because of similarities in overall organization, we conclude that the molluscan metanephridial system is homologous with that of the annelids not only at the cellular but also at the organ level.

## Background

Body cavities together with the functionally related nephridial systems are among the most discussed character complexes in the organization of molluscs [1,2]. Within bilaterians (lophotrochozoans in particular) two main types can be recognized: (1) Acoelomate and pseudocoelomate conditions are correlated with protonephridia. Here ciliary activity of the terminal cell (cyrtocyte or solenocyte) causes ultrafiltration from the primary body cavity through a basal membrane and an ultrafiltration weir into the lumen of the protonephridial duct, which releases the primary filtrate (often with modification) to the external environment. (2) In coelomate animals ultrafiltration is mainly mediated by contractions of muscles (located at the walls of the vascular system - even inside of "podocytes" [3]) from the remainders of the primary body cavity (often "blood vessels") into the secondary body cavity. The so-called metanephridia are the releasing tubes, which are often provided with a ciliary funnel at the distal end and usually modify the primary urine. Contrary to protonephridia the locations of ultrafiltration site and modification tube are largely separated.

While there is wide agreement on the homology of protonephridia (e.g. [4]), there are opposing concepts on the homology of metanephridia (metanephridial systems). Firstly, there is the question of the homology between protonephridia and metanephridial systems. Based on the functional continuum between the two systems [2,5], Haszprunar [6] suggested a direct homology of protonephridial and metanephridial ultrafiltration cells, i.e. the terminal cell with the podocyte. The second question concerns the homology between metanephridial systems of different phyla: Here most authors favour homoplasy [4,7-12]. This particularly applies to the Mollusca, where a homology of their metanephridia with those of other lophotrochozoans, such as annelids, had been rejected mainly because of differences in development [12,13].

During the course of development, molluscs typically exhibit both types of excretory systems, protonephridia and a metanephridial system (Figure 1 of [14]). Polyplacophorans have always played a central role in considerations on molluscan phylogeny. Because they exhibit many plesiomorphic traits, they often were placed at or near the base of the Mollusca [11,15,16]. Accordingly, data on Polyplacophora seem particularly important for comparison with other phyla. New data on metanephridial system formation in Polyplacophorans are required, since previous studies were carried out with inadequate methods [17] or are incomplete [13]. In a previous study we have investigated differentiation and organization of the protonephridia of the polyplacophoran *Lepidochitona corrugata*[14]. In this study we continue with the metanephridial system of this species.

**Figure 1:**
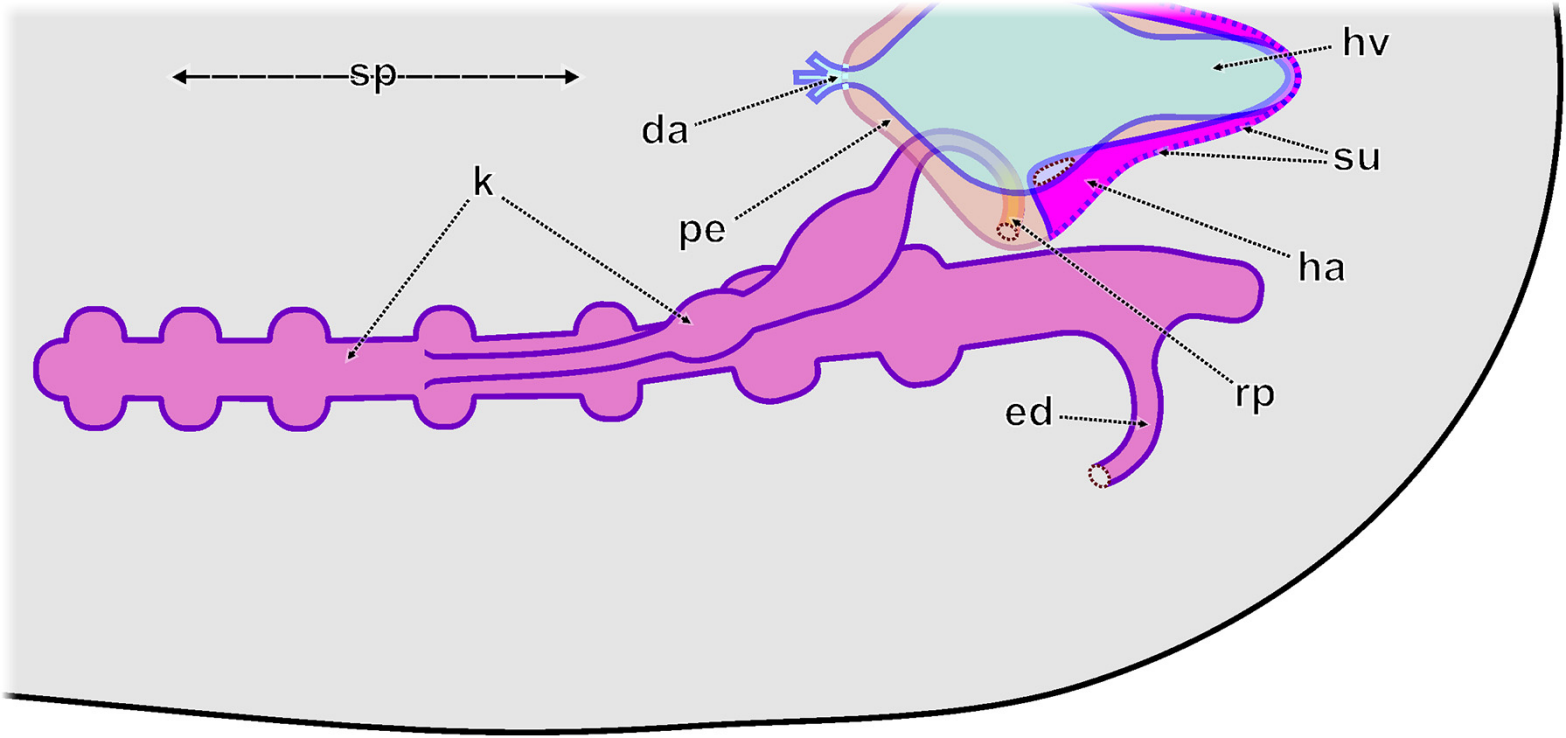
**Schematic representation of an adult (fully developed) metanephridial system plus the pericard/heart complex of a polyplacophoran similar to that of a member of the genus *Lepidochitona.*** Dorsal view of the left side. da, dorsal aorta; ed, efferent nephroduct; ha, heart atrium; hv, heart ventricle; k, kidney; pe, pericardium; rp, renopericardial duct; sp, sagittal plane; su, sites of ultrafiltration.

The fully developed metanephridial excretory system of polyplacophorans, such as *Lepidochitona corrugata,* is situated at the posterior region of the animal (Figure 1). It is composed of the heart being surrounded by the pericardium, paired renopericardial ducts, paired kidneys and paired efferent nephroducts. Accordingly, the excretory and the pericard/heart complex are closely intertwined in structure and function. As reviewed by Morse & Reynolds [18] ultrafiltration takes place from the haemocoel of the heart atria into the pericardial lumen by podocytes which are part of the epithelium of the pericardium covering the walls of the atria [19,20]. The ultrafiltrate (i.e. the primary urine) becomes transported by heart activity or by ciliary beat in the renopericardial ducts from the pericardial lumen towards the kidneys. Here the primary urine becomes modified by reabsorption and drained off by the efferent nephroducts and the kidney opening into the mantle groove.

## Results

### Overview of development

In *Lepidochitona corrugata* the development of the metanephridial system is a rapid process. It starts soon after settlement, when the animals have reached a length of about 0.4 mm, and is largely completed at a length of about 0.62 mm with achievement of full functionality. Thereafter, mainly increase in size takes place by allometric growth. The development of the metanephridia and the central pericard/heart complex occurs almost simultaneously, thus the processes leading to the formation of individual components are interlocked. Individual processes proceed continuously until finished, for better understanding alone we categorize four successive stages: (1) Appearance of an initial coelomic anlage that gives rise to all major components of the metanephridial system; (2) differentiation of this common anlage to the unpaired anlage of pericardium plus heart and to the paired anlagen of renopericardial ducts, kidneys and efferent nephroducts; (3) achievement of functional nephridial organization; (4) final differentiation towards adult organization.

### Stage 1 - initial anlage (spms 3, 3a, 4)

**Figure 2:**
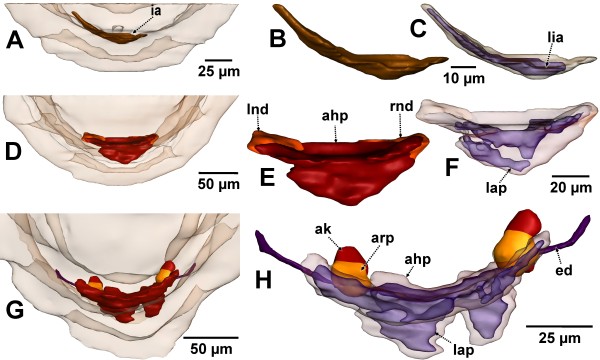
**Stages of metanephridial system development in *Lepidochitona corrugata *(surface renderings)**. **A, D, G.** Posterior part of the body from dorsal (external surface transparent). **B, C, E, F, H.** Details of developing metanephridial systems enlarged. **A–C**. spm 3a. **D–F**. spm 4. **G–H**. spm 5. **C, F, H**. External surface transparent with internal lumen visible. ahp, anlage of heart and pericardium; ak, anlage of the kidney; arp, anlage of the renopericardial duct; ed, efferent nephroduct; ia, initial anlage of metanephridial and central blood vessel system; lap, lumen of the anlage of pericardium and heart; lia, lumen of the initial anlage; lnd, left nephridial differentiation; rnd, right nephridial differentiation.

The initial anlage of the metanephridial system and coelomic components arises in the most posterior embayment of the perivisceral cavity, dorsal and slightly posterior of the anal opening, resting atop the commissure of the lateral nerve cords. In the earliest stage, where it could be identified (spm 3), it comprises an unpaired, irregularly shaped small cell cluster. In a slightly larger specimen (spm 3a) the anlage became distinctly enlarged and achieved a cap-shaped appearance sitting posteriorly adjacent to the most posterior gut loop (Figure 2A-C). It has achieved an endothelial organization containing an internal homogeneous cavity. The endothelial lining is mostly thin with thickenings at the sites of nuclei.

**Figure 3:**
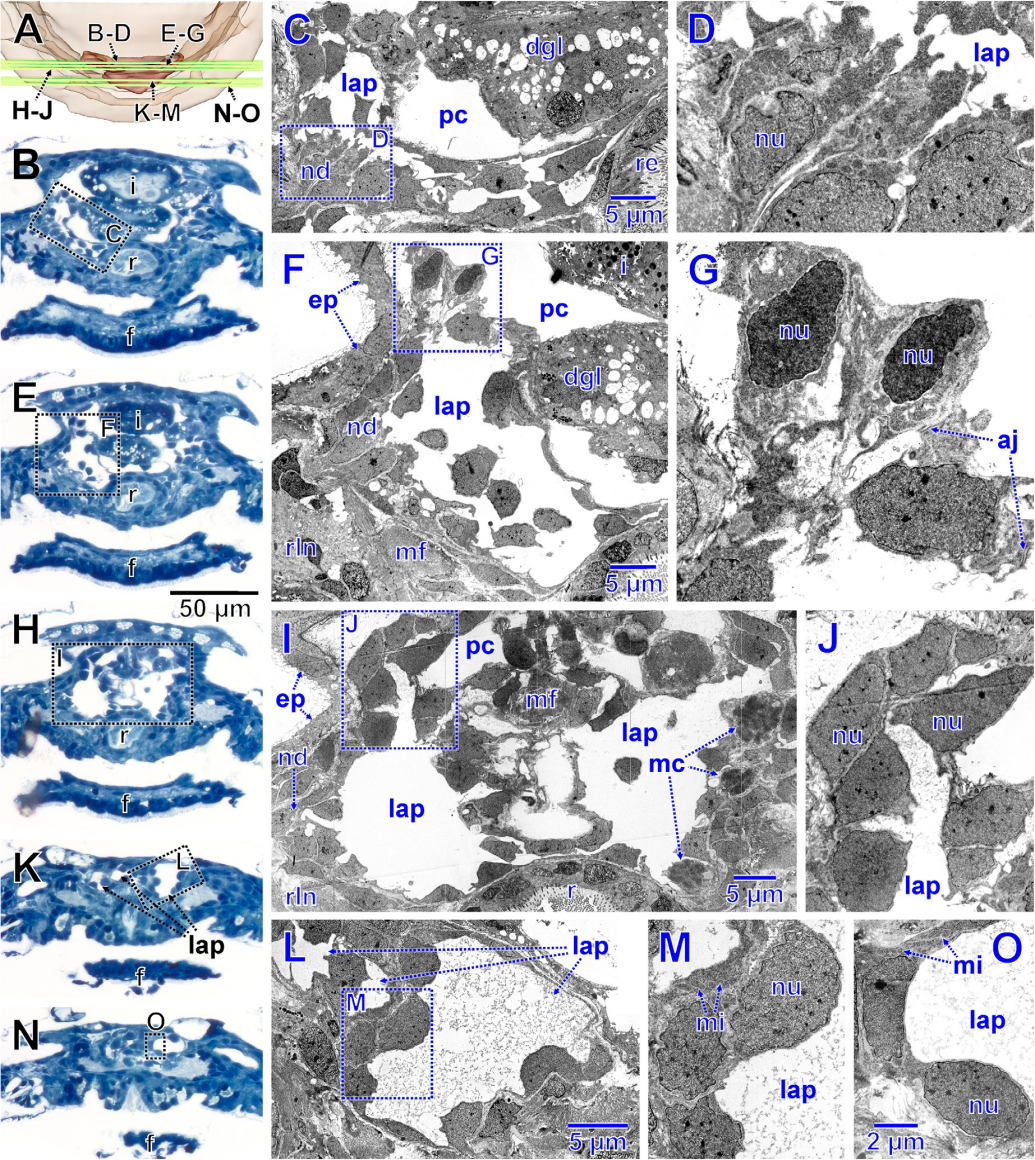
**Stage of metanephridial system development in *Lepidochitona corrugata *(spm 4). A.** Posterior part from dorsal with transparent external surface and planes (ortho slices) of section images shown in B-O. **B, E, H, K, N.** Total LM cross sections through the anlagen of the metanephridial and pericard/heart complex with stippled rectangles indicating the position of details given in the TEM sections C, D, F, G, I, J, L, M, O. **C.** Right side of the anlage with early lateral primordium of the kidney (enlarged in D). **F.** Right side of the anlage slightly further posterior than C, dorsal differentiation enlarged in G. **I.** Right side of the anlage slightly further posterior than F, dorsal dense tissue enlarged in J. **L, M, O.** Posterior portion of the anlage. aj, adhaerens junction; dgl, digestive gland; ep, epidermis; f, foot; i, intestine; lap, lumen of the anlage of pericardium and heart; mc, mitotic cells; mf, muscle fibers; mi, mitochondria; nd, nephridial differentiation; nu, nucleus; pc, primary body cavity; r, rectum; rln, right lateral nerve cord.

In a subsequent stage (spm 4) the anlage (Figures 2D–F, 3) has become differentiated in several ways. The lumen of the entire anlage is no longer homogeneous, but is divided into several pouches (Figure 3K, L) by in-growing endothelial parts in the posterior region. The in-growing tissue appears to represent the precursor of the heart. The endothelium still is thin with cell extensions as well as nuclei (Figure 3L, M) protruding into the lumen. Most of the volume of the endothelial cells is occupied by the nuclei. The cytoplasm is very dense, containing numerous mitochondria (Figure 3M, O), many of which exhibit an elongate shape. The basement lamina of the endothelium is a very thin layer of extracellular matrix, which is hardly discernable from the cytoplasm of the endothelial cells. The apical parts of the endothelial cells are interconnected by adhaerens junctions (Figure 3G). The anlage as a whole also shows considerable mitotic activity *in situ* (Figure 3I). Paired extensions located latero-anteriorly (Figure 2E, F) with densely packed nuclei (Figure 3C, D, F) are particularly remarkable. The position of these extensions correspond perfectly to the anlagen of the nephridial components in subsequent development stages, accordingly they are interpreted to represent first differentiations of renopericardial duct and kidney. Ciliation could not be detected yet. However, in the region of these primordia there are some structures that resemble centrioles of cilia; these might represent anlagen of the cilia of the future renopericardial duct. Like in previous and subsequent stages the anlage is located adjacent to the posterior body wall. Since the latter is mainly formed by musculature, the surrounding tissue largely consists of muscle fibers (Figure 3F, I).

### Stage 2 – differentiation of the nephridial components (spms 5, 6, 7)

**Figure 4:**
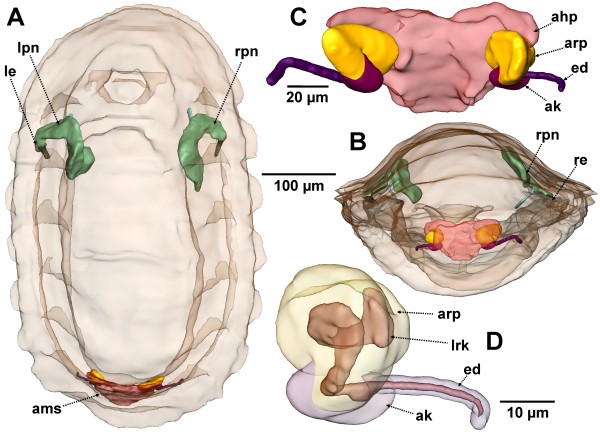
**Stage of metanephridial system development in *Lepidochitona corrugata *with individual components differentiated (spm 7, surface renderings and interactive 3D model). A, B.** Total specimen from dorsal and posterior (external surface transparent). **C.** Anlage of the metanephridial system and pericard/heart complex from anterior**. D.** Left nephridial components with external surface transparent and internal lumen from anterior. ahp, anlage of heart and pericardium; ak, anlage of the kidney; ams, anlage of the metanephridia and pericard/heart complex; arp, anlage of the renopericardial duct; ed, efferent nephroduct; le, left eye; lpn, left protonephridium; lrk, lumen of the renopericardial duct and kidney anlage; re, right eye; rpn, right protonephridium. **The interactive 3D-model** can be accessed by clicking onto Figure 4 in Additional file 1. Use interactive tools in Adobe Reader to rotate and zoom the image or highlight selected portions.

**Figure 5:**
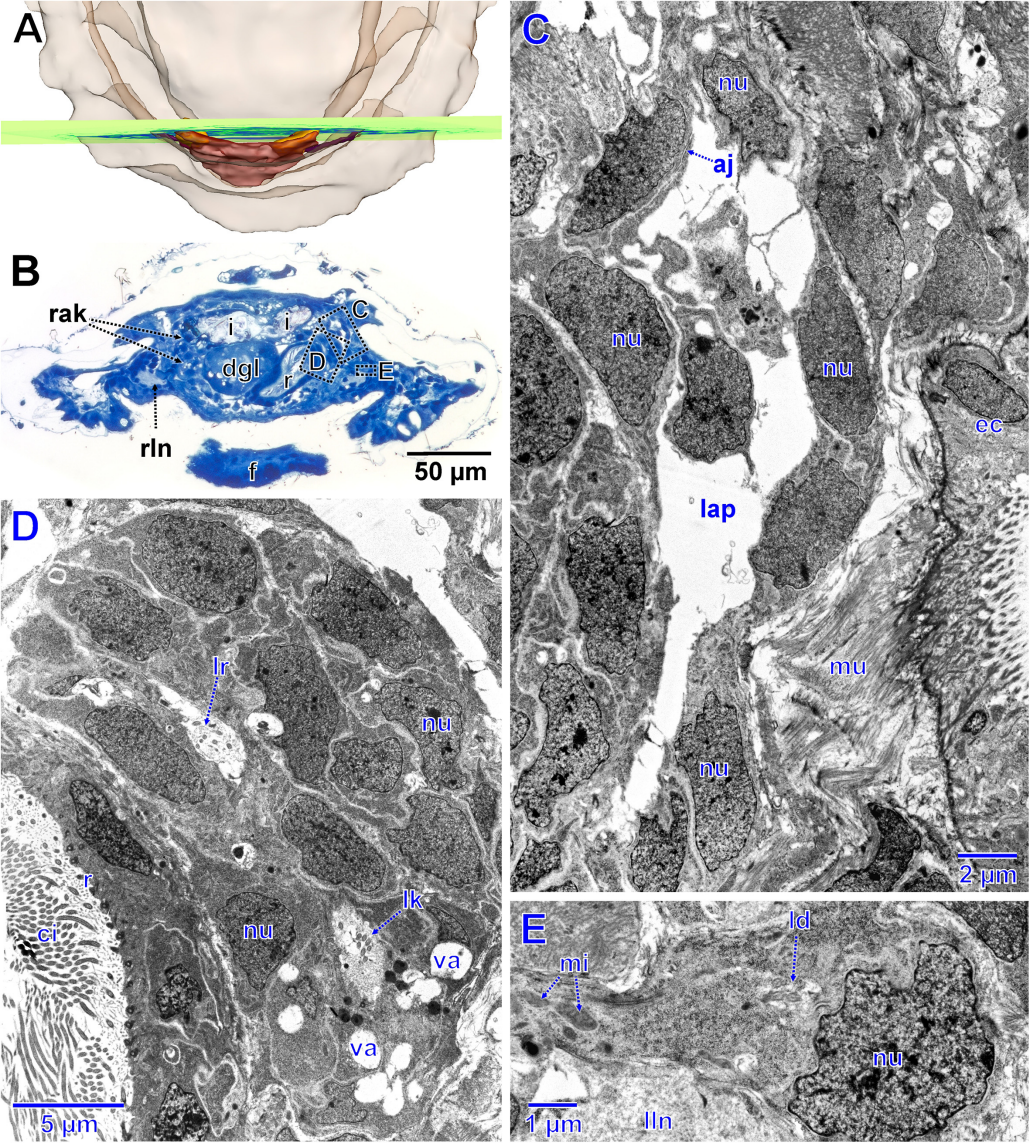
**Stage of metanephridial system development in *Lepidochitona corrugata *with individual components differentiated (spm 7).A.** Posterior part of specimen from dorsal with transparent external surface and plane (ortho slice) of section images shown in B–E. **B.** Total LM cross section through the anlagen of the metanephridial system with stippled rectangles indicating the position of details given in the TEM sections C–E. **C.** Extension of the anlage of heart and pericardium on left side forming the connection to the renopericardial duct. **D.** Left anlage of renopericardial duct (dorsal) and kidney (ventral). **E.** Left efferent nephroduct. aj, adhaerens junction; ci, cilia; dgl, digestive gland; ec, epidermal cell; f, foot; i, intestine; lap, lumen of the anlage of pericardium and heart; ld, lumen of the efferent duct with ciliation; lk, lumen of the kidney anlage with ciliation; lln, left lateral nerve cord; lr, lumen of the renopericardial duct anlage with ciliation; mi, mitochondria; mu, muscle fibers; nu, nucleus; rak, right anlage of renopericardial duct and kidney; r, rectum; rln, right lateral nerve cord; va, vacuole.

**Figure 6:**
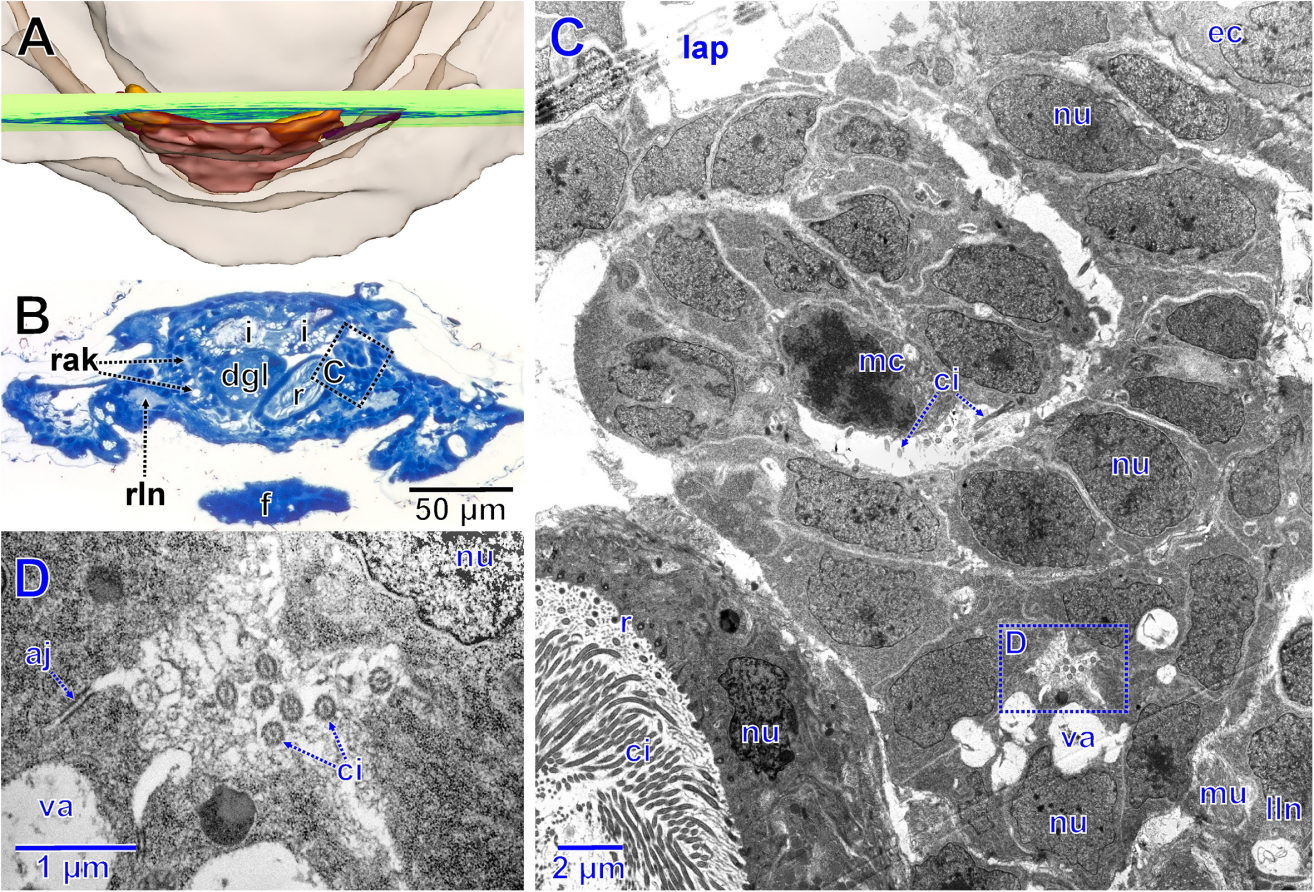
**Stage of metanephridial system development in *Lepidochitona corrugata *with individual components differentiated (spm 7).A**. Posterior part of specimen from dorsal with transparent external surface and plane (ortho slice) of section images shown in B–D. **B**. Total LM cross section through the anlagen of the metanephridial system with stippled rectangle indicating the position of details given in the TEM sections C, D. **C.** Left anlage of renopericardial duct (dorsal) and kidney (ventral) showing the connection of the lumen of the renopericardial duct with that of the anlage of pericardium and heart. Stippled rectangle indicating the position of the TEM section D. **D.** Lumen of the anlage of the kidney. aj, adhaerens junction; ci, cilia; dgl, digestive gland; ec, epidermal cell; f, foot; i, intestine; lap, lumen of the anlage of pericardium and heart; lln, left lateral nerve cord; mc, mitotic cell; mu, muscle fibers; nu, nucleus; rak, right anlage of renopericardial duct and kidney; r, rectum; rln, right lateral nerve cord; va, vacuole.

The anlagen of the renopericardial ducts, kidneys and efferent nephroducts have become clearly separated from the medially located anlage of heart and pericardium (Figures 4, 5A, 6A). The latter anlage has become more voluminous; it still shows out-bulges separated by external infoldings in the posterior region. Like in earlier stages, the endothelial lining is very thin, except for the sites of the nuclei (Figure 5C).

The anlagen of the remaining components of the metanephridial system are antero-laterally attached to the anlage of heart and pericardium (Figures 4A, B, C, 5A, 6A). There are three regions distinguishable in each of the paired structures (Figure 4C, D): The most conspicuous one is placed adjacent to the pericardial anlage, which is followed by a somewhat smaller middle portion and a thin elongate tube leading laterally above the lateral nerve cord towards the mantle groove. These three parts correspond to the future renopericardial duct, the kidney and the efferent nephroduct. From their origin in the antero-ventro-lateral wall of the pericardium and heart anlage, the anlage of the renopericardial duct extends anteriorly for a short distance and then bends posteriorly again. It merges ventro-posteriorly with the anlage of the kidney; the anlage of the efferent nephroduct emerges from the kidney anlage laterally. The efferent nephroduct runs laterally towards the mantle groove, tightly dorsally surrounding the lateral nerve cord (Figure 4C, D) and showing a blind end in a short distance from the body surface. All portions contain an internal narrow lumen (Figures 4D, 5C–E, 6C, D) that is continuous with that of the pericardial and heart anlage (Figure 6C). The lumen of the efferent nephroduct is extremely narrow (Figure 5E) and shows some cilia (Figures 5D, E, 6C, D). The ciliary bases are continuously distributed all over the length of the internal surface of the entire nephridial anlage from the opening into the pericardial anlage to the end of the efferent nephroduct. In cross sections the number of cilia ranges between eight (Figure 5D) and three (Figure 5E) with the number decreasing towards the distal end of the efferent nephroduct. At this stage all cells of the nephridial anlagen are connected by apical adhaerens junctions next to the lumen. All components are distinctly different from the pericardium and heart anlage in histology. The anlagen of the renopericardial duct and kidney are conspicuous, while the one of the efferent nephroduct forms a thin hollow tissue extension. The tissue of the prospective renopericardial duct is very dense with large cuboid cells and little granular cell plasma containing few mitochondria in addition to the densely arranged nuclei (Figures 5D, 6C). The cells of the kidney primordium are somewhat smaller. At this stage they already exhibit vacuoles (Figures 5D, 6C), which constitutes a first sign of differentiation towards future kidney histology. Mitotic activity is still evident in the anlagen (Figure 6C).

### Stage 3 – achievement of nephridial organization (spms 8, 9)

**Figure 7:**
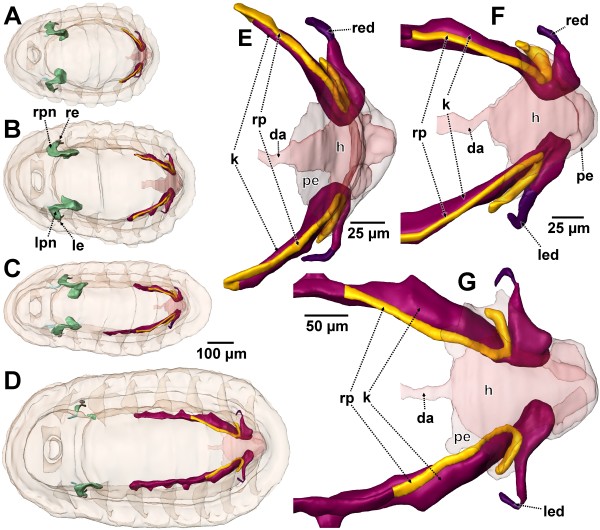
**Later stages of metanephridial system development in *Lepidochitona corrugata *(surface renderings). A–D.** Total specimens, scale bar for all between C and D. **A**. spm 8. **B**. spm 10. **C**. spm 12. **D**. spm 14. **E–G**. Metanephridial systems enlarged with transparent pericardial wall and heart. **E**. spm 8. **F**. spm 12. **G**. spm 14. da, dorsal aorta; h, heart; k, kidney; le, left eye; led, left efferent nephroduct; lpn, left protonephridium; pe, pericardium; re, right eye; red, right efferent nephroduct; rp, renopericardial duct; rpn, right protonephridium.

When the animals have reached a length of approximately 620 μm all functional elements of the metanephridial system are essentially completed. The structural details closely resemble those of the adult animal. The relative dimensions, however, still differ significantly (Figure 7).

**Figure 8:**
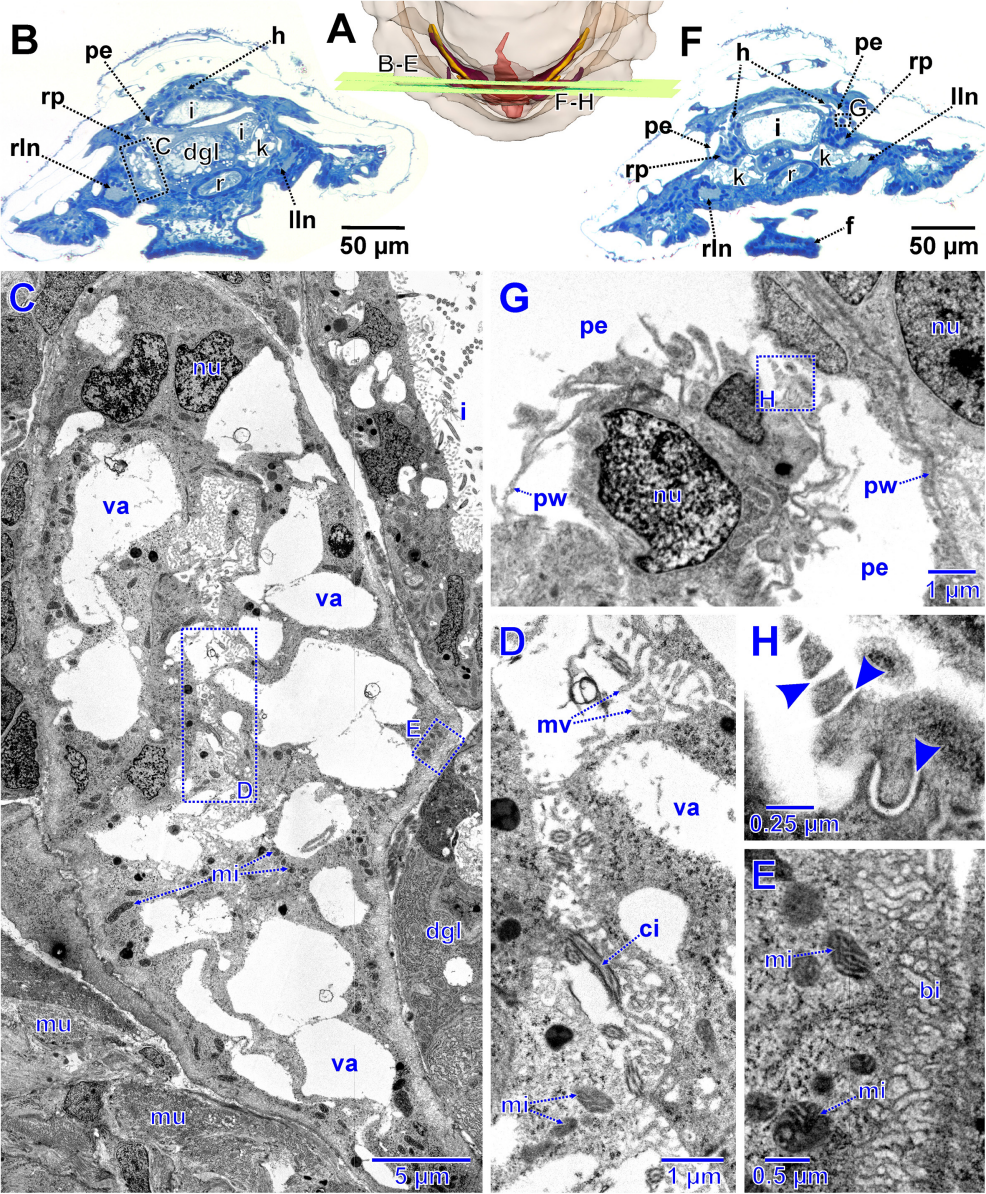
**Advanced stage of metanephridial system development in *Lepidochitona corrugata *(spm 8). A.** Posterior part of specimen from dorsal with transparent external surface and planes (ortho slices) of section images shown in B–H. **B.** Total LM cross section through the anlagen of the metanephridial system with stippled rectangles indicating the position of details given in the TEM sections C–E. **C.** Right metanephridial kidney. Stippled rectangles mark areas shown in D and E. **D.** Metanephridial lumen. **E.** Surface of kidney with basal infoldings. **F.** Total LM cross section through the anlagen of the metanephridial system with stippled rectangles indicating the position of details given in the TEM sections G and H. **G.** Ultrafiltration site in the atrial wall. Stippled rectangle marks area shown in H. **H.** Ultrafiltration site, arrowheads indicating ultrafiltration slits. bi, basal infoldings; ci, cilium; dgl, digestive gland; f, foot; h, heart; i, intestine; k, kidney; lln, left lateral nerve cord; mi, mitochondria; mu, muscle fibers; mv, microvilli; nu, nucleus; pe, pericardium; pw, pericardial wall; r, rectum; rln, right lateral nerve cord; rp, renopericardial duct; va, vacuole.

**Figure 9:**
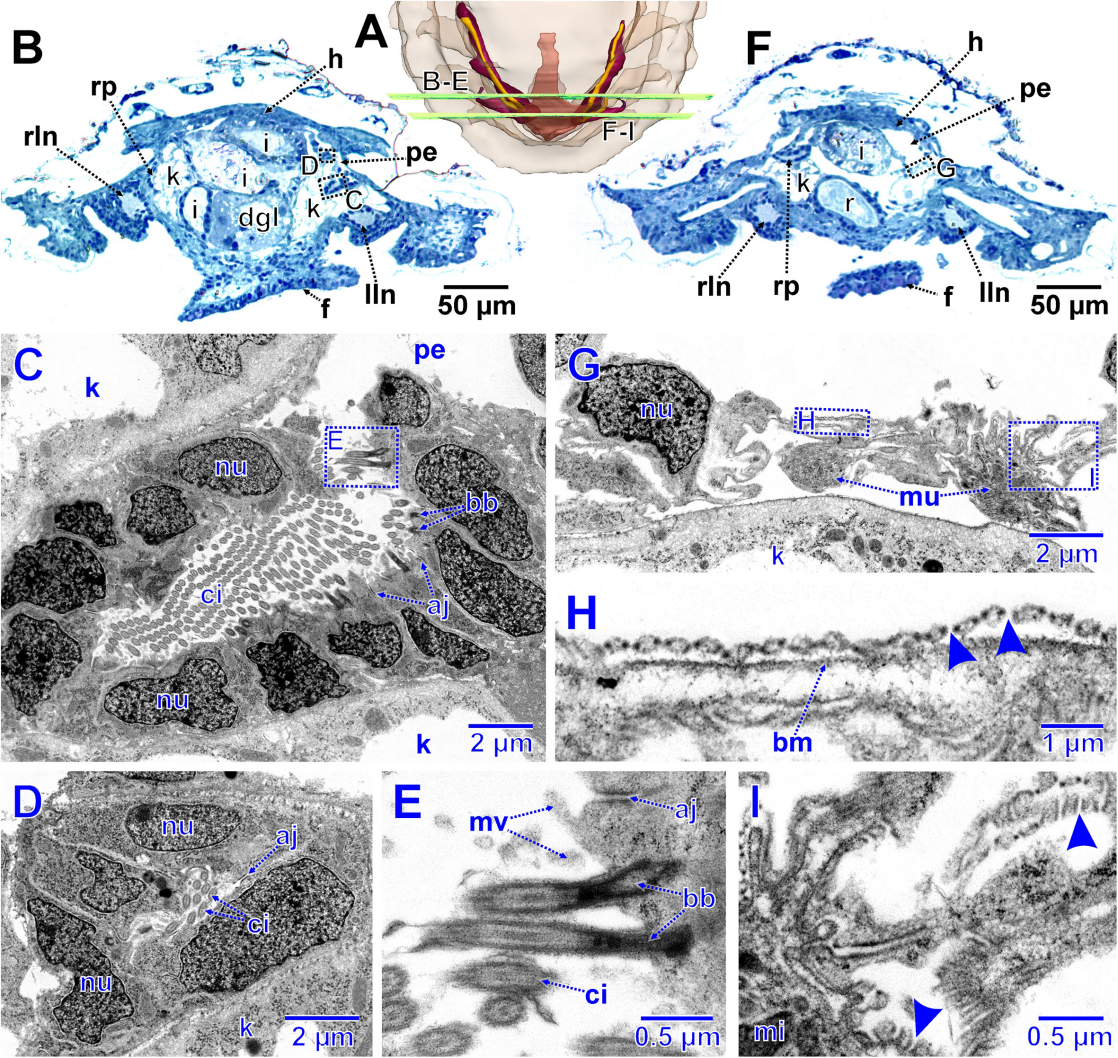
**Advanced stage of metanephridial system development in *Lepidochitona corrugata *(spm 10). A**. Posterior part of specimen from dorsal with transparent external surface and planes (ortho slices) of section images shown in B–I. **B**. Total LM cross section through the anlagen of the metanephridial system with stippled rectangles indicating the position of details given in the TEM sections C and D. **C.** Opening of the renopericardial duct into the pericardium on left side. Stippled rectangle marks area shown in E. **D.** Left renopericardial duct. **E.** Cilia originating from wall of renopericardial duct. **F**. Total LM cross section through the anlagen of the metanephridial system with stippled rectangle indicating the position of details given in the TEM sections G–I. **G**. Ventral, left pericardial wall near the base of the atrium sitting atop the kidney. Stippled rectangles mark areas shown in H and I. **H, I.** Ultrafiltration sites, arrowheads indicate ultrafiltration slits. aj, adhaerens junction; bb, basal body; bm, basal membrane; ci, cilia; dgl, digestive gland; f, foot; h, heart, i, intestine; k, kidney; lln, left lateral nerve cord; mi, mitochondrium; mu, muscle fibers; mv, microvilli; nu, nucleus; pe, pericardium; r, rectum; rln, right lateral nerve cord; rp, renopericardial duct.

The pericardium is trapezoid in shape. The pericardial cavity is enclosed by a thin endothelium (Figure 8G); inside the heart is differentiated (Figures 7A, E, 8A, B, F). It consists of a median portion, the ventricle that shows an anterior elongation, the prospective aorta. The ventricular wall is conspicuous and partly formed by muscle fibers. Latero-posteriorly the ventricle is continuous with paired strings of fine tissue that extends towards the base of the pericardium, presenting the auricles of the heart. At the base of this tissue near the pericardial wall, cell groups protruding into the pericardial lumen can be found (Figure 8G). These cells are podocytes and already exhibit ultrafiltration sites, which in principal organization remain the same during development. A single podocyte could be detected which bears a cilium protruding into the pericardial lumen. The podocytes exhibit a folded, enlarged surface with filtration slits between pedicles (Figure 8H). The slits have a width of 20–30 nm and a meandering pathway. The pedicles have electron dense thickenings next to the slits. There is a basal lamina (same as in a slightly larger specimen shown in Figure 9H) underlying the ultrafiltration site towards the primary body cavity.

Near the region of the ultrafiltration sites, the renopericardial duct emerges from the pericardium (Figure 8B). From this site each duct bends backwards and after a short distance turns for- and outwards. It then turns down- and backwards before it enters the kidney (Figure 7E). In this development stage the duct comprises a narrow tube with a narrow lumen. Cilia are originating from the entire inner surface; all these cilia extend towards the kidney. In cross sections of the duct the number of cilia is about 50 and thus highest next to the pericardium and decreases subsequently to four in proximity to the kidney. The diameter also decreases with distance from the pericardium (external diameter near the pericardium: 14 μm, near the kidney: 6 μm; lumen width near the pericardium: 5 μm, near the kidney 2 μm).

The kidney itself is an elongate organ. It extends from the junction with the renopericardial duct, underneath and adjacent to the latter posteriorly towards the heart, where it bends laterally until it merges into the efferent nephroduct. It is relatively voluminous and slightly laterally compressed (height x width maximum: 50 x 20 μm). The epithelium of the kidney is composed of large cells with capacious vacuoles with a diameter up to 10 μm (Figure 8C). The remaining cytoplasm is very dense and contains numerous often elongate mitochondria. In addition, there is an abundance of dark, roundish vesicles of varying size and unknown nature (Figure 8C, D). Internally the kidney contains a narrow indented lumen (Figure 8C, D). Numerous microvilli protrude from the inner cell surfaces into this lumen. In addition few cilia can be found. Most of the cilia originate from the renopericardial duct cells, but some also from the kidney cells. The entire basal (external) portion of the kidney cells bears a dense basal infolding system (thickness up to 1 μm); the basement membrane of the kidney cells forms numerous infoldings (Figure 8C, E) resembling a microvillous border.

The efferent nephroduct extends laterally from the kidney towards the mantle groove (Figure 7A, E). It is a simple tube with thin walls. In cross sections it is composed of three to four cells. The most proximal part was mostly found widened (lumen diameter up to 10 μm) and may function for temporary urine storage. The lumen in the remaining portion is very narrow to as low as 2 μm near the nephropore. The cells of the efferent nephroduct are ciliated on the inside. The number of cilia in cross sections increases towards the nephropore (from four to ten). All cilia extend towards the nephropore; some ciliary tips protrude into the mantle groove. The cells, which form the nephropore, differ from the surrounding epidermal cells by their cell plasma, which is distinctly less electron dense.

Except for the heart, which has mesenchymate organization, the entire tissue of the metanephridial system has endothelial character. All cells are apically interconnected by adhaerens junctions.

### Stage 4 – final differentiation towards adult organization (spms 10–16)

After a length of 620 μm had been reached development of the metanephridial system primarily consists of reinforcement of filtration and reabsorptive structures. Most obvious is the size increase of nephridial structures relative to the remaining body of the animal by allometric growth (Figure 7B–D, F, G).

In a 753 μm long specimen (spm 10) the ciliation of the renopericardial duct has distinctly been enforced. There are nearly 250 cilia in the renopericardial duct next to the opening in the pericardium visible (Figure 9C). The ultrafiltration sites at the base of the atria (Figure 9G–I) are much more conspicuous.

Particularly evident during further development is the growth of the kidneys. This can take place asymmetrically (Figure 7C). In specimen with 1.159 mm (spm 14) body length both kidneys already extend further anteriorly than to the middle of the animal (Figure 7D). The kidneys also start forming out-pouchings. In addition, the transition of the renopericardial ducts towards the kidneys becomes strongly shifted during development (Figure 7A–D). At a length of 621 μm the renopericardial ducts run forwards and turn backwards before entering the kidney. By elongation of the kidney the site of transition becomes shifted to the dorsal side. During further development the relative length of the renopericardial ducts is decreasing, since growth of the kidneys continues while the renopericardial ducts hardly grow anymore.

**Figure 10:**
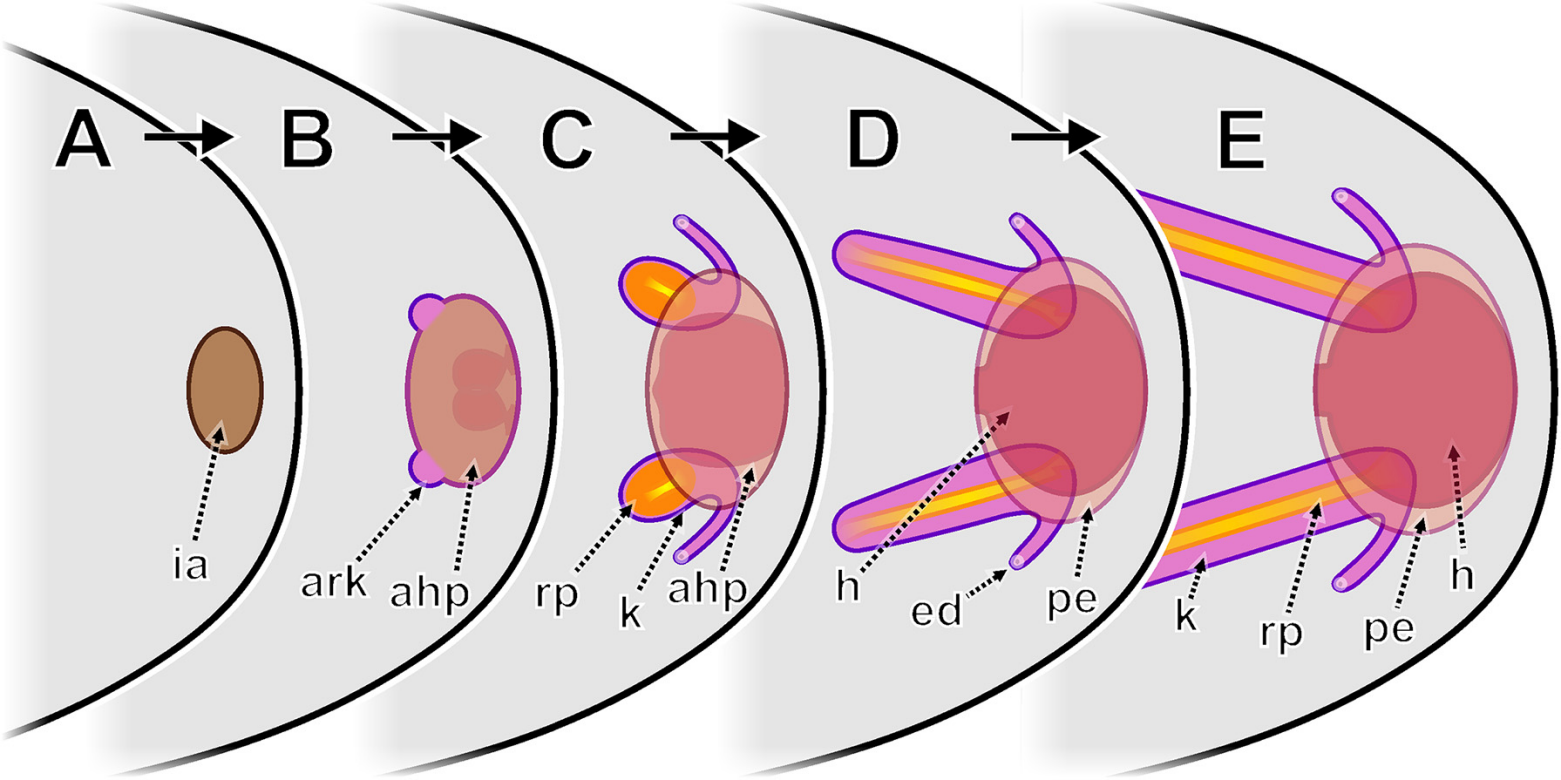
**Schematic representations of stages during formation of the metanephridial system in *Lepidochitona corrugata.*A**. Initial anlage appearing. **B**. Kidney components become separated from the common anlage, first differentiations of the heart appear in remaining anlage. **C**. Further differentiation of kidney components and heart. **D**. Basic filtration organization completed, kidneys become enlarged, heart formation finished. **E**. Additional substantial growth of the kidneys. ahp, anlage of heart and pericardium; ark, anlage of renopericardial duct and kidney; ed, efferent nephroduct; h, heart; ia, initial anlage of the metanephridial and circulatory system; k, kidney; pe, pericardium; rp, renopericardial duct.

At the end of these processes the excretory system essentially has reached the adult condition. All major processes leading to this condition are concisely presented in Figure 10.

## Discussion

### Comparison with previous data on molluscan nephrogenesis

Previous reports on nephrogenesis are restricted to only four of eight molluscan classes: Gastropoda, Bivalvia, Cephalopoda and Polyplacophora.

Data on Gastropoda are scarce and differ considerably: The anlagen of kidneys and pericardia are reported to be paired (e.g. [21]: *Patella*) or unpaired ( [22]: *Marisa*), to be separate (e.g. [23]: *Viviparus*) or common (e.g. [22,24]: *Marisa*). The descriptions agree on the fact, that initially solid anlagen next to the hindgut give rise to kidney and pericardium. In gastropods the renopericardial complex is differentiated prior to metamorphosis [21,25,26].

Renopericardial development of Bivalvia has been investigated by a number of studies, but they date back between 80 and 130 years (e.g. [27-32]). These studies can be summarized as follows: Paired, solid anlagen give rise to kidneys, pericardium and (later) gonads. Kidney differentiation with formation of a lumen precedes that of the heart; the kidney becomes tube-like and gets in contact with the epidermis and, thereby, forms a porus to the outside; most of the remaining initial anlage gives rise to the prospective pericardium; the two sides subsequently fuse in the sagittal plane to form the unpaired pericardium and heart.

Cephalopoda show a highly derived mode of development. Data on renopericardial ontogeny are limited to *Sepia, Loligo* and *Octopus*[33-37], where kidney development with formation of a lumen distinctly precedes that of the pericardium. From the onset of pericardium differentiation, there is a detectable connection to the kidney, which becomes the future renopericardial duct.

Information about nephrogenesis of the Polyplacophora is scarce. In a study on general development of *Acanthochiton discrepans* Hammarsten and Runnström [17] report the kidneys to be formed as outgrowth of the preformed pericardium; they subsequently get in contact with the epidermis and form a nephroporus. The more recent studies by Bartolomaeus [38] and Salvini-Plawen & Bartolomaeus [13] on *Lepidochitona cinerea* added ultrastructural details on pericardium and heart development. In this study observations are restricted to three stages (250, 550 and 1000 μm length). Whereas we can confirm the principle mode of development in *Lepidochitona* (i.e. outgrowth of the releasing duct from the pericardium with minimal support from the ectoderm), we note a number of differences in the details of nephrogenesis: Concerning the first anlage at about 550 μm body length, Bartolomaeus [38] and Salvini-Plawen & Bartolomaeus [13] described a paired anlage of the pericardium epithelial tubes left and right from the rectum with already differentiated podocytes and cilia. In contrast our study shows an unpaired mass of cells which differentiate towards an epithelial cavity lacking ultrafiltration cells (podocytes). In a specimen with about 1 mm body length Bartolomaeus described a large pericardium with heart and caudally emerging pericardioducts with blind endings in the body wall. In our specimens of this size the whole metanephridial system is fully developed and shows nephropores, and the pericardioducts emerge relatively far anterior on the sides of the pericardium.

Both species, *Lepidochitona cinereus* and *L. corrugata*, are closely related, nevertheless heterochronic effects, i.e. shifts in the timing of nephrogenesis, cannot be fully excluded. On the other hand we based our study on 16 specimens of varying stages of nephrogenesis, whereas the former study investigated specimens of different stages only. Accordingly, studies on the nephrogenesis of further species, in particular of representatives of the Lepidopleurida, appear necessary to proceed towards inference of a ground pattern of nephrogenesis in the Polyplacophora.

### Homology considerations

General aspects:

Inference of homology always is a matter of probabilities and even identical genetic background of a phenotypic subject does not solve the matter i.e. "homocracy" structures; [39]. All homologization is comparison, and various hierarchical levels need to be clearly distinguished [40,41]. Concerning the excretory systems the levels to be considered are; (1) Iterative homology of ultrafiltration cells, i.e. the protonephridial cyrtocyte with the metanephridial podocyte; (2) Serial homology of larval protonephridia with adult metanephridial system; (3) Supraspecific homology of both systems among the Mollusca and beyond. Current understanding of the modular organization of the genome makes it possible to consider homologies (similarities based on shared gene expression) independently from synapomorphies (appearance of characters at the phylogenetic tree).

Proto- and metanephridial system in *Lepidochitona corrugata*:

The (developing) metanephridial system resembles the protonephridia [14] of *Lepidochitona corrugata* in several aspects: (1) Both organ systems start most internally with ultrafiltration sites. These are followed by a transportation duct, then a modification portion, and finally an efferent duct, which opens via a pore to the outside. Nematodes or arthropods have principally different excretory structures, so this similarity is not trivial. (2) In both cases epithelial ultrafiltration cells filter molecules from the primary (haemocoel) to a secondary (endothelial) body cavity. (3) The fine structure of the ultrafilter itself is identical and complex; it is composed of meandering slits, which form gaps between pedicle elements, and these gaps are interconnected by a thin diaphragm. (4) The transportation duct following the ultrafiltration portion shows dense downstream ciliation. (5) Both organs exhibit an absorptive portion, the kidney, which has identical cellular organization with basal infoldings, apical microvillous border and strongly vacuolized cytoplasm. Again the case of vertebrate kidney shows that this similarity is not trivial.

The two systems also exhibit differences:

(1) Ultrafiltration of protonephridia is done by terminal cells, while it is carried out by podocytes in the case of metanephridia. Essentially, the difference between these two cell types is presence (terminal cells) and absence (podocytes) of ciliation. Nevertheless, a homology between these cell types appears likely [6], since intermediate forms have been reported, e.g. the "cyrtopodocytes" in the amphioxus *Branchiostomma* and do occur also in early juvenile bivalves (Ruthensteiner et al. unpubl.). In addition, both types do occur subsequently during ontogeny in certain polychaetes (e.g. [42]). The finding of a cilium in a podocyte of *Lepidochitona corrugata* – remarkably the first report of a podocyte bearing a cilium in a mollusc – lends substantial support to this concept.

(2) In the metanephridial system filtration pressure is (partly) generated by heart beat, while it is (partly) generated by the ciliation of terminal cells in protonephridia. However, this difference seems to be of limited significance, since in both systems a major part of negative pressure generation is facilitated by the same structures, the downstream ciliation of the excretory duct (see [43,44] for review of molluscan metanephridial systems).

In conclusion, we assume that there is substantial homology between the protonephridial and metanephridial system in *Lepidochitona* at least at the cellular level. For *Lepidochitona* it seems possible that the metanephridial system as a whole is a serial homologue of the protonephridial system. However, the overall data are still fragmentary and do not permit a final clarification of that question. For example, it remains unclear if the absorptive portions (kidneys) of protonephridia are homologous throughout the Mollusca [14].

Proto- and metanephridial systems in molluscs and related taxa:

The current evolutionary understanding of nephridial systems in the Mollusca is as follows:

(1) Protonephridia: There is wide agreement that a single pair of anteriorly placed, larval protonephridia ("head-kidneys" sensu [45], "archinephridia" sensu [46] of polychaetes) is a synapomorphic organ system at least for Trochozoa (i.e. Entoprocta, Mollusca, Sipuncula, and Annelida; doubtfully also Nemertinea) [4,5,9,47] and thus plesiomorphic for the Mollusca (see also [14]).

(2) Metanephridial system: Salvini-Plawen (e.g. [11-13,48-50]) considered metanephridial nephroducts ("kidneys") to be evolved within the Mollusca at the evolutionary level of Testaria (Polyplacophora and Conchifera). These "emunctoria" are interpreted as specialized portions of previously undifferentiated "pericardioducts", which originally lack a function in excretion. Salvini-Plawen provides two main reasons for his hypothesis: (a) the absence of an ultrafiltration/reabsorption (metanephridial) system in aplacophoran molluscs; (b) the non-homology with excretory organs of other phyla, such as the metanephridia of annelids. However, both arguments seem doubtful: (ad a) TEM findings suggest regular metanephridial function also among aplacophorans: Podocytes were described from the pericardium of both aplacophoran taxa, Solenogastres [51] and Caudofoveata [52], providing evidence for ultrafiltration. Morphological evidence for reabsorption has been reported in the caudofoveate *Falcidens crossotus*: Cells of the lower pericardial ducts (releasing also the gametes) exhibit all ultrastructural characteristics of absorptive cells including a distinct basal infolding system (Figure 23 G of [53]). Accordingly, a metanephridial system probably also exists in Solenogastres. (ad b) Salvini-Plawen (e.g. [12]) and Salvini-Plawen & Bartolomaeus ( [13], also [38]) also rejected a homology between molluscan and annelid/sipunculan metanephridia by assumed different germ layer origin and different mode of formation: While the molluscan kidneys are a mesodermal outgrowth of the pericardium, the annelid kidneys are formed as ingrowth of ectodermal epidermis. However, there are molluscs showing metanephridial anlagen without a connection to a pericardium [54] and there are certain annelids (e.g. [55,56]) where metanephridia descend from the coelothelium of the previously formed coelomic pouches (see also [57]). Thus, because of the variability in nephrogenesis of both, Mollusca and Annelida this reasoning cannot be upheld. Also the second argument against homology of molluscan and annelid nephridia is weak at least: Molluscs lack a funnel at the beginning of the nephroduct as found in annelids [12]. However, a funnel is just an outbulging opening of the metanephridial duct into a voluminous coelomic cavity. Again, annelids vary in this respect, and particularly those with a restricted coelomic cavity, such as some leeches, lack a funnel too [58].

Accordingly, and based on substantial similarities in fine-structure, we think that a metanephridial system consisting of terminal podocytes at the pericardial walls (ultrafiltration site), a renopericardial duct (transport and generation of pressure by cilia), and a nephroduct ("kidneys": reabsorption) belongs to the basic pattern of the Mollusca and is homologous with those of other trochozoan phyla.

Admittedly, the data basis for these assumptions is still poor. Further comprehensive studies with a 4-dimensional approach (3D-analysis of successive development stages) on nephridial organs of more molluscan taxa and other invertebrates are required for a better understanding of the questions on homology and synapomorphy raised above and thus on the framework of evolution of filtration excretory organs among Metazoa.

## Conclusions

The metanephridial system of the polyplacophoran *Lepidochitona corrugata* is formed from a coelomic anlage that gives also rise to the central pericard/heart complex. Lateral outgrowths of this anlage form the kidneys and nephroducts that get in contact with the body surface. Because of similarities in overall organization and because of the variability of nephrogenesis in both Mollusca and Annelida, we conclude that the molluscan metanephridial system is homologous with that of the annelids not only at the cellular but also at the organ level.

During development and in adult organization the metanephridial system of the polyplacophoran *Lepidochitona corrugata* shares many detailed traits with the protonephridia of the same species. In both cases, there are most distal ultrafiltration sites; downstream they are followed by a duct supplied with reabsorptive portions (kidneys) and an efferent duct leading towards the body surface. The histology of individual portions is identical. This suggests there is a serial homology of various cell types and between the two excretory systems. It might even be argued that there is the same kind of homology between the two organ systems as a whole.

## Methods

Juvenile specimens of the chiton *Lepidochitona corrugata* (Reeve, 1848) were collected near the Observatoire Océanologique de Banyuls-sur-Mer (France). Details for specimen acquisition and the subsequent fixation, embedding and light microscopical (LM) procedures were provided previously [14,59].

Table 1 shows details and methods applied for all specimens used. Seventeen specimens (all except spm 11) were completely serially cross-sectioned for LM with a diamond knife and a section thickness of 1.5 μm. Specimen 11 was alternately sectioned to LM and ultrathin sections (10–20 ultrathin sections in between two LM sections). In the other specimens utilized for transmission electron microscopy (TEM) analysis in addition to LM sectioning procedures were as follows: single LM sections of special interest were chosen, cut out of the section ribbons, detached from the slide, remounted on a resin block and re-sectioned for TEM (10–15 ultrathin sections from a 1.5 μm thick LM section).

**Table 1:** Specimens investigated with methods applied in addition to LM serial section examination

**Specimen number (spm)**	**Specimen length**	**Embedding medium**	**Section thickness**	**TEM**	**Number of LM sections used for subsequent TEM sectioning**	**Computerized 3D analysis (Amira software)**
3	415 μm	Spurr’s lvr	1.5 μm	no	-	SR, total specimen
3a	504 μm	Spurr’s lvr	1.5 μm	no	-	SR, metanephridial region
4	513 μm	Spurr’s lvr	1.5 μm	yes	10	SR, metanephridial region
4a	522 μm	Agar lvr	1.5 μm	yes	2	aligned 3D stack
4b	558 μm	Agar lvr	1.5 μm		-	-
5	579 μm	Spurr’s lvr	1.5 μm	no	-	SR, total specimen
6	580 μm	Spurr’s lvr	1.5 μm	yes	12	SR, metanephridial region
7	604.5 μm	Spurr’s lvr	1.5 μm	yes	17	SR, total specimen
7a	594 μm	Spurr’s lvr	1.0 μm	no	-	aligned 3D stack
8	621 μm	Spurr’s lvr	1.5 μm	yes	14	SR, total specimen
9	652.5 μm	Spurr’s lvr	1.5 μm	yes	7	aligned 3D stack
10	753 μm	Spurr’s lvr	1.5 μm	yes	17	SR, total specimen
11	~800 μm	Spurr’s lvr	1.5 μm / 1.0 μm	yes	-	-
			50–70 nm			
12	852 μm	Spurr’s lvr	1.5 μm	no	-	SR, total specimen
13	886.5 μm	Spurr’s lvr	1.5 μm	yes	12	SR, metanephridial region
14	1159.5 μm	Spurr’s lvr	1.5 μm	no	-	SR, total specimen
15	1465.5 μm	Agar lvr	1.5 μm	yes	2	-
16	1821 μm	Agar lvr	1.5 μm	no	-	-

Length specifications refer to embedded condition; lvr, low viscosity resin; SR, surface rendering.

Protocols for TEM analysis as well as 3D-analysis and -visualization were also provided previously [14,59]. Preparation of the interactive 3D-model (Additional file 1) essentially followed the procedures outlined in Ruthensteiner & Heß [60].

## Competing interests

The authors declare that they have no competing interests.

## Authors’ contributions

BR and NB designed the research. Laboratory procedures, data acquisition and analysis were mainly carried out by NB. All authors contributed to drafting, and read and approved the final manuscript.

## Supplementary Material

Additional file 1 **3D model of Figure 4.** By clicking in Adobe Reader anywhere onto the figure the 3D model of the juvenile ***Lepidochitona corrugata*** can be interactively accessed. Click here for file
