# Supplementary figures and images for: Development of the excretory system in a polyplacophoran mollusc: stages in metanephridial system development
Source: Front Zool. 2012 Sep 14;9:23. doi: 10.1186/1742-9994-9-23 (PMC3494531; doi:10.1186/1742-9994-9-23)

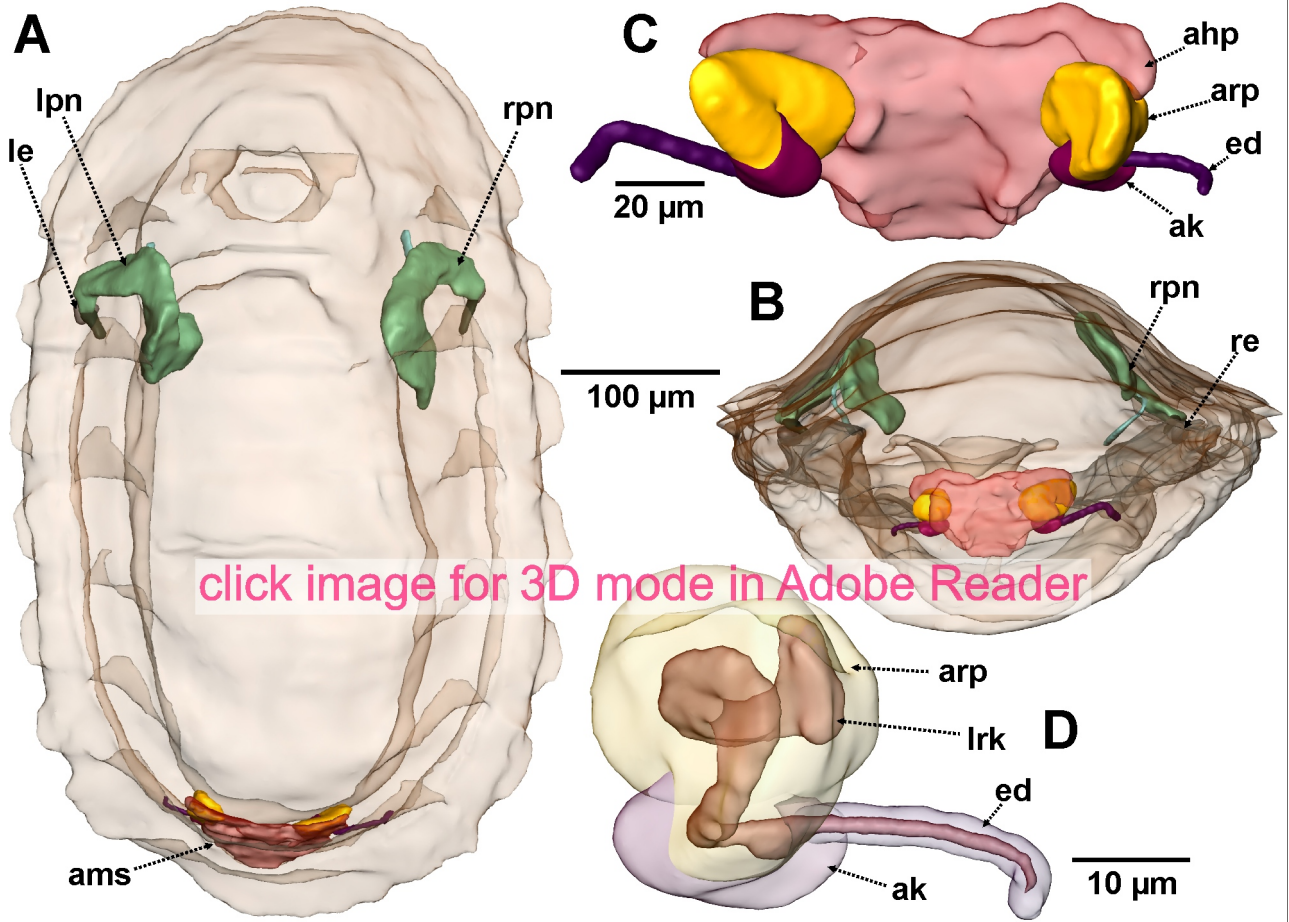

Supplement: Additional file 1 — 3D model of Figure 4. By clicking in Adobe Reader anywhere onto the figure the 3D model of the juvenile Lepidochitona corrugata can be interactively accessed. [file 1742-9994-9-23-S1.pdf]
